# Clinical advantage of zero to minimal fluoroscopy for cardiac electronic device implantable

**DOI:** 10.1002/joa3.12988

**Published:** 2024-01-03

**Authors:** Naoya Kataoka, Teruhiko Imamura

**Affiliations:** ^1^ Second Department of Internal Medicine University of Toyama Toyama Japan

To editor:

The recent advent of the zero to minimal fluoroscopy (ZMF) approach in clinical practice aims to curtail radiation exposure from fluoroscopy and obviate the need for contrast agent usage. Luke et al., through a systematic literature review, demonstrated that ZMF yielded analogous success rates, procedural durations, and overall complication rates for permanent cardiac implantable electronic device (CIED) implantation, while markedly reducing fluoroscopy time and radiation exposure when juxtaposed with the traditional fluoroscopy method.^1^

Nonetheless, several concerns have surfaced regarding the ZMF approach. Its purported advantage lies in abstaining from fluoroscopy,^1^ yet it frequently relies on alternative modalities such as three‐dimensional mapping systems. An imperative requisite is the conduction of cost‐effectiveness analyses, employing measures like Quality‐Adjusted Life Years, to ascertain the comparative efficacy between these methodologies. Notably, the extant fluoroscopy method stands as an entrenched procedure with sparse complications and minimal radiation exposure, rendering the clinical implications of adopting ZMF in the contemporary landscape ambiguous.

The rationalization behind ZMF exhibiting abbreviated fluoroscopy duration and reduced complication rates versus the conventional fluoroscopy method remains enigmatic. For instance, in prior investigations concerning cardiac resynchronization therapy device implantation, the ZMF approach necessitated an added femoral vein puncture for three‐dimensional mapping.^2^ Consequently, ZMF may harbor the potential for prolonged procedural durations and elevated complication rates.

While the ZMF approach circumvents the use of contrast agents,^1^ it is noteworthy that CIED implantation typically necessitates a mere 10–20 mL of contrast agent. Importantly, intravenous administration of contrast agents seldom incites contrast‐induced nephropathy.^3^

## CONFLICT OF INTEREST STATEMENT

Authors declare no conflict of interests for this article.
